# Does Multiple Capitals Disclosure Affect the Capital Market? An Empirical Analysis in an Integrated Reporting Perspective

**DOI:** 10.3389/fpsyg.2022.837209

**Published:** 2022-02-24

**Authors:** Yanqi Sun, Xin Qiao, Yi An, Qiaoling Fang, Na Wu

**Affiliations:** ^1^School of Economics and Management, Beijing Institute of Petrochemical Technology, Beijing, China; ^2^Shandong Office, China Banking and Insurance Regulatory Commission, Jinan, China; ^3^School of Management, Ocean University of China, Qingdao, China; ^4^College of Management, Ocean University of China, Qingdao, China; ^5^School of Accounting, Tianjin University of Finance and Economics, Tianjin, China

**Keywords:** integrated reporting, content analysis, firm value, decision making, investor, capital market

## Abstract

Integrated reporting (IR), as a novel corporate reporting approach, focuses on how six forms of capital promote corporate value. This paper explores whether this kind of multiple capitals disclosure (MCD) framework has an impact on the capital market. Using a sample of Chinese A-share firms from 2012 to 2016, we examine the relationship between MCD quality and firm value. The results indicate that a higher MCD quality leads to a greater firm value. Our results are robust to a variety of sensitivity tests. Further evidence suggests that MCD quality could increase profitability by affecting the decision-making of non-financial stakeholders and enhance the value relevance of financial information by affecting the decision-making of investors. The paper helps understand how the IR approach affects the perception of investors on the value of a firm. The findings of the paper are of interest to academics, corporate management, investors, and governmental officials.

## Introduction

Financial reporting and sustainability reporting are two major strands of current corporate reporting, but their isolated focuses limit their bigger role in helping decision-making of investors (Frías-Aceituno et al., 2013; Lodhia, 2015; Robertson and Samy, 2015). Traditional financial reporting does not capture the impact of non-financial operations and performances, such as many sorts of intangible capital, on financial performances. Although some non-financial aspects may be addressed by CSR reporting, these aspects are disconnected from the firm’s financial performance (Barth et al., 2017). By emphasizing the integration of financial information and non-financial information, integrated reporting (IR) is often perceived to be a potentially useful tool to overcome such limitations of prior corporate reporting (Reimsbach et al., 2017). Such a major change in the information disclosure enables possibilities to better communicate with stakeholders, providing them with a holistic view of an organization concerning how multiple capitals of an organization contribute to value creation over time (Gutiérrez-Goiria et al., 2021).

The International Integrated Reporting Council (IIRC), established in 2010, is a pioneering international organization that focuses on accelerating the adoption of integrated reporting and integrated thinking within mainstream business entities across the world (Liu et al., 2019; Beyne et al., 2021). The IIRC developed the International Integrated Reporting Framework (IIRF) in that it identifies “Six Capitals,” comprising financial capital, manufactured capital, human capital, social capital, intellectual capital, and natural capital, which are viewed as inputs and outputs of a company for value creation (IIRC, 2013). Thus, the IIRF provides multi-capital guidance for corporate reporting. Doni et al. (2019, p. 6) conclude that “the IIRC’s multiple capitals framework, with its six forms of capital, is an innovation in corporate financial and non-financial reporting.” Flower (2015) acknowledges that the main value of the IIRF is its capability to allow managers to concentrate more on the integrated management of the six forms of capital. According to Silvestri et al. (2017, p. 7), IR is “a more effective reporting approach because it focuses on value creation through the lens of the six forms of capital … rather than sustainability reporting’s focus on environmental and social impacts through the lens of stakeholder materiality.” IR is expected to promote sustainable business practices (Argento et al., 2019), and to help create a more sustainable world (Steyn, 2014).

As an innovative form of corporate reporting, IR has influenced reporting practices by companies in many countries (De Villiers et al., 2014a; Stubbs and Higgins, 2018; Abhayawansa et al., 2019). For instance, in South Africa, it is mandatory that all companies listed on the Johannesburg Stock Exchange are required to implement IR on a “apply or explain” basis from March 2010, based upon the King III Report on Corporate Governance for South Africa (known as King III) (De Villiers et al., 2014a; Cortesi and Vena, 2019). Later on, With the publication of the King IV Report on Corporate Governance for South Africa (known as King IV) in 2016, the “apply or explain” requirement has been replaced with the “apply and explain” basis (Willows and Rockey, 2018; Le Roux and Pretorius, 2019). The Securities and Exchange Commission of India also urges the top 500 Indian companies to apply the IR approach to prepare for their annual reports (Barth et al., 2017). As for China, although there are currently no mandatory requirements for companies for the adoption of IR, the Chinese government has realized the importance of IR for corporate reporting (Barth et al., 2017). China’s Ministry of Finance has joined the IIRC and conveyed support for IR in its recently published five-year plan (IIRC, 2018). It believes that IR is consistent with the trend of corporate reporting reform toward global and domestic sustainable development and encourages Chinese companies to apply IR to fulfill the sustainable development agenda (Yang et al., 2012).

Integrated reporting focuses on how six forms of capital promote firm value (Anifowose et al., 2020). In fact, disclosure about the six capitals can be informative to investors. According to behavioral theory, as investors have limited capability of attention and processing information, only enhancing the salience of information can attract investors’ attention and make the information processing more easily (Barth et al., 2017). IR increases the salience of information by providing investors with information concerning how multiple capitals affect firm value. Specifically, by interconnecting the financial aspect with manufactured, intellectual, human, social and relational, and natural aspects of a firm and analyzing how all these aspects relate to firm value, IR depicts a holistic picture of the firm’s ability to create value (Barth et al., 2017; Zhou et al., 2017). A positive link between IR disclosure practices and firm value has been identified in prior South African studies (Lee and Yeo, 2016; Barth et al., 2017). A qualitative study conducted by Atkins and Maroun (2015) show that South African institutional investors see IR as an improvement on the conventional annual reports. As Rinaldi et al. (2018) suggest exploring the effects of IR in different contexts, we raise the question of whether the IR approach affects the pricing decision making of investors also hold true in China.

This research aims at offering empirical evidence to answer the question of whether and how the IR approach creates value for investors in China. In this paper, we develop an IR quality index on the basis of the multiple capitals framework of the IIRF, as an instrument for content analysis to assess multiple capitals disclosure (MCD) quality^1^ by Chinese listed companies. We examine the relationship between MCD quality and firm value. The results reveal that a higher MCD quality leads to a greater firm value. We further find that the MCD quality affects the decision-making of non-financial stakeholders and investors, respectively.

The contribution that our research intends to provide to the extant academic literature is threefold. First, this paper makes a contribution to the growing literature on the relevance of IR to the capital market. Whereas previous studies mainly focus on the South African context, we rely on a Chinese sample to derive interesting insights on the much-debated nature of IR. The current research offers empirical evidence from a seldom-studied setting. The decision-usefulness of IR is often questioned (Slack and Tsalavoutas, 2018; Mans-Kemp and van der Lugt, 2020). If it can be verified that the IR approach is useful for the decision-making of investors and non-financial stakeholders, a key objective of the IIRF can be achieved (IIRC, 2013). Second, there is little research on how MCD affects the capital market. Previous studies either focus on the content elements of IIRF (Lee and Yeo, 2016; Zhou et al., 2017) or concern single capital, such as intellectual capital in Salvi et al. (2021a) and human capital in Salvi et al. (2021b). Our study comprehensively considers the MCD framework derived from IIRF. Third, whether an organization uses the IR approach depends on IR’s ability to shape stakeholders’ perceptions of the organization. In other words, the decision of an organization to use the IR approach is substantively affected by the perceived benefit provided by IR to stakeholders. We attempt to investigate the two channels of the market effect through which the IR approach enhances firm value. One is how the MCD quality affects the decision-making of non-financial stakeholders and the other one is how the MCD quality affects the decision-making of investors. Thus, this study contributes to behavioral decision theory (Velte and Stawinoga, 2016).

The remainder of our paper is organized as follows. Section “Literature Review” reviews relevant literature and section “Hypothesis Development” develops the research hypotheses. Section “Materials and Methods” describes the research method for this study. Section “Results” presents and discusses the research findings. Section “Discussion and Conclusion” concludes the paper.

## Literature Review

Prior studies have examined the effect of IR (the adoption of IR or IR quality) on firm value. Lee and Yeo (2016) employ the South African dataset to assess the relationship between IR quality and firm valuation after the adoption of IR in the country, and they observe a positive association between the two variables, particularly for those companies with higher organizational complexity or higher external financing needs. In a similar vein, Barth et al. (2017) investigate the associations between IR disclosure quality of South African companies and corresponding market reactions in the period 2011–2014. The results indicate that higher IR disclosure quality (annual rankings of integrated reports according to EY Excellence in IR awards) results in higher firm value. In addition, Pavlopoulos et al. (2019) survey the relationship between IR quality and firm value, using an international dataset in that the sample companies adopt IR for corporate reporting. They obtain similar findings with previous studies and suggest that the adoption of IR would bring benefits to firms. Based on an international sample consisting of 110 companies, Salvi et al. (2021a) finds a significantly positive relationship between structural, human, social and relational capitals disclosure quality and firm value. Similarly, Salvi et al. (2021b) documents that human capital disclosure quality has a significant and positive impact on firm value.

However, the findings of prior studies are not conclusive in the international setting. Wahl et al. (2020) gauge whether investors benefit from the adoption of IR using an international sample of 167 listed companies. They do not find evidence that the adoption of IR has a significant effect on firm value. In a similar vein, Hsiao et al. (2021) examine whether the voluntary adoption of IR has an impact on firm value using a sample of global firms, but no strong evidence is found. Their study suggests that IR adoption cannot provide additional benefits for improving firm value in a voluntary adoption setting.

Through this literature survey on prior studies, it can be summarized that although some studies have examined the impact of the IR approach on firm value in South African and international settings, these findings of prior literature may not be generalizable to other countries. Thus, it is interesting to investigate whether the IR approach has an impact on firm value in China.

## Hypothesis Development

Based on the Discounted Cash Flow model, the firm value is calculated by discounting expected future cash flows to their present value by applying the cost of capital rate. Therefore, a firm’s value could be affected by the cost of capital and the expected cash flows (Wahl et al., 2020). Specifically, if the cost of capital of a firm can be reduced and its expected cash flows can be increased, the market value of the firm rises. Several channels through which IR can reduce the cost of capital were identified by previous studies (Barth et al., 2017; Wahl et al., 2020; Salvi et al., 2021a,b). The first is that IR can provide high-quality information and thus reduce information asymmetry between principles and agents. Secondly, IR allows investors to access the holistic picture of a firm in an easy way, leading to a larger investor base. Thirdly, according to signaling theory, firms adopt IR approach (a signal to the market) to distinguish themselves from the other firms with low transparency. Thus, when a firm adopts IR approach, investors bid a higher price for the shares of the firm. Fourthly, the adoption of IR is associated with a lower risk premium. One perspective is that the adoption of IR is a way to obtain organizational legitimacy in society. A firm that adopts IR approach reduces its risk of losing organizational legitimacy. The other perspective is that IR elaborates risks a firm confronts and how its strategy and business model deal with those risks, thereby leading to a reduction in the estimated information risk.

There are two channels through which IR affects the expected cash flows: the real effect and the market effect (Barth et al., 2017). A real effect relates to improved internal decisions of the firm that generate higher future cash flows. According to Mio et al. (2021, p. 6), “IR can lead to integrated thinking and integrated decision making, e.g., by breaking down silos and focusing on long-term, instead of short-term, strategy that results in better real decisions and enhanced firm value”. Based on a scenario-based experiment, the study of Esch et al. (2019) show that integrated reporting is important for organizations as it promotes internal decision making. A market channel relates to an improved information environment for outside capital providers. In such an environment, investors can estimate future cash flows more accurately and non-financial stakeholders are willing to contribute more to the development of the firm (Barth et al., 2017; Esch et al., 2019; Flores et al., 2019).

Prior research has provided empirical evidence for this respect. Lee and Yeo (2016) observe that IR quality positively impacts firm value in the light of capital markets and accounting performance. In line with Lee and Yeo (2016), Barth et al. (2017) also find a positive association between annual rankings of integrated reports according to EY Excellence in IR awards and firm value. More recently, Pavlopoulos et al. (2019) claim that there is a positive relationship between IR quality and the market valuation of a firm. Salvi et al. (2021a,b) provide evidence confirming that both intellectual capital disclosure quality and human capital disclosure quality have a positive impact on firm value. On the basis of the above discussion, we postulate that:

*Hypothesis 1:* There is a positive association between IR quality by Chinese firms and firm value.

## Materials and Methods

### Sample Selection and Data Source

In this study, 247 A-share listed companies in the environmentally sensitive sectors were selected as a sample since companies in these industrial sectors were very sensitive to the environment, and they usually disclosed more non-financial information such as sustainability-related information in their annual financial reports and annual non-financial reports, so as to legitimize their status to be a responsible organization in society. Annual financial reports and annual non-financial reports (e.g., sustainability reports or CSR reports) of sample companies from 2012 to 2016 were the data source for this study.^2^ Data for IR quality by sample companies were hand-collected by a researcher involved in this study, while the financial data were obtained from the China Stock Market and Accounting Research (CSMAR) database. Special treatment (ST) companies^3^ were removed, and the companies which lacked financial data were excluded as well. Of the total of 1,087 firm observations, 64 are from the petroleum industry, 810 are from the chemistry industry, and 213 are from the plastic industry. The average market capitalization of sample firms is 9,397.48 million Chinese Yuan with minimum and maximum values varying between 623.91 and 100,291 million Chinese Yuan, respectively.

### Content Analysis

Content analysis of corporate reports was adopted as the research method to gauge IR quality by sample companies, as it is often used as well as considered to be an appropriate method in the area (i.e., Setia et al., 2015; Zinsou, 2018; Liu et al., 2019). As IR is based on the integration of six forms of capital (Nicolò et al., 2021), a MCD quality index was developed on the basis of six forms of capital of IIRF for content analysis (refer to Table 1), which is in line with Melloni (2015) and Demartini and Trucco (2017). The MCD framework has been receiving a growing recognition from scholars (Gleeson-White, 2015; Ahmed Haji and Anifowose, 2016).

**TABLE 1:** The IR quality index.

Capital Type	Description	Score Criterion
1. Financial capital	The pool of funds available to an organization for use in the production of goods or the provision of services, obtained through financing, such as debt, equity or grants, or generated through operations or investments	The score of 3: a numerical and detailed described disclosure item The score of 2: a numerical but not detailed described disclosure item The score of 1: a generally described disclosure item The score of 0: no disclosures
2. Manufactured capital	Manufactured physical objects (as distinct from natural physical objects) available to an organization for use in the production of goods or the provision of services, including buildings and equipment infrastructure (such as roads, ports, bridges and waste and water treatment plants)	
3. Intellectual capital	Organizational, knowledge-based intangibles, including intellectual property, such as patents, copyrights, software, rights and licenses; and organizational capital such as tacit knowledge, systems, procedures and protocols	
4. Human capita	People’s competencies, capabilities and experience and their motivations to innovate, including their alignment with and support for an organization’s governance framework, risk management approach and ethical values; ability to understand, develop and implement an organization’s strategy; and loyalties and motivations for improving processes, goods and services, including their ability to lead, manage and collaborate	
5. Social and relationship capital	The institutions and the relationships within and between communities, groups of stakeholders and other networks, and the ability to share information to enhance individual and collective well-being. Social and relationship capital includes shared norms and common values and behaviors; key stakeholder relationships and the trust and willingness to engage that an organization has developed and strives to build and protect with external stakeholders; intangibles associated with the brand and reputation that an organization has developed; reputation that an organization has developed and an organization’s social license to operate	
6. Natural capital	All renewable and non-renewable environmental resources and processes that provide goods or services that support the past, current or future prosperity of an organization, including air, water, land, minerals and forests, as well as biodiversity and ecosystem health	

A scoring system with a four-point scale (0–3) was then applied to assess MCD quality. The coder scored the MCD quality from 0 to 3 based on the corporate report against the quality criteria set in Table 1. The maximum score of a form of one capital is “3.” Therefore, a firm could obtain a quality score ranging from 0 (minimum score) to 18 (=3 × 6, maximum score).

Before the coding of corporate reports, two researchers of this paper involved in this study were trained for the data collection on the basis of the developed MCD quality index. Then we conducted a pilot test for 10 reports amongst the reports so as to improve the reliability of the coding. If there were any divergencies, a discussion would be made until a consensus was achieved. Then, another round of pilot test for 10 new reports was carried out by the two researchers. Results obtained were then compared using SPSS macro for calculating a Krippendorff’s α coefficient of each comparison. The results showed that Krippendorff’s α coefficients were 0.8869. 0.8661, 0.8548, 0.9153, 0.9436, 0.9458, 0.8725, 0.8660, 0.9475, and 0.8743, all exceeding the threshold of 0.80. This confirms the reliability of the coding (Melloni, 2015). After the pilot test, one scorer did the formal content analysis for all corporate reports.

### Variables and Model

The dependent variables, independent variables, and control variables for this study are demonstrated in Table 2. The dependent variable was the firm value that was measured by Tobin’s Q in terms of market performance. By referring to some previous studies (i.e., Lee and Yeo, 2016; Pavlopoulos et al., 2019), we identified seven control variables, including board size, ownership concentration, company size, ownership type, leverage, corporate growth, and CEO duality. In addition, we controlled the year fixed effect.

**TABLE 2:** Definition of variables.

Variable	Definition
**Dependent variables**	
Firm value (TobinQ)	The market value of a company divided by total assets at the end of year
**Independent variables**	
MCD quality score	The quality of multiple capitals disclosure of each company
**Control variables**	
Board size (Board)	The total number of directors in the board
Ownership concentration (Share)	The shareholding ratio of the largest shareholder
Company size (Size)	The natural logarithm of a firm’s total assets
Ownership type (SOE)	A dummy variable: 1 for a firm is state-owned, 0 otherwise
Leverage (Lev)	Total liabilities divided by total assets at the end of each year
Company growth rate (Growth)	The rate of operating income growth
CEO duality (CEO-dual)	A dummy variable: 1 for a firm whose chairman of the board and the CEO are the same person, 0 otherwise
Year fixed effects	A dummy variable: 1 for the current year, 0 otherwise

In order to examine the relationship between MCD quality and firm value from various perspectives, the OLS regression model was performed as follows:

TobinQ _i,__t+1_ = α_0_ + α_1_MCD _i, t_ + α_2_ Board _i,t_ + α_3_ Share _i, t_ + α_4_ Size _i,__t_ + α_5_ SOE _i,__t_ + α_6_Lev _i,__t_ + α_7_ Growth _i,__t_ + α_8_ CEO-dual _i,t_ + YearDum + θ _i,__t_

We used a lead-lag approach by one year for the dependent variable, as adopted by Dhaliwal et al. (2011), to address endogeneity concerns caused by reverse causality and simultaneity.

## Results

### Descriptive Statistics and Correlation Analysis

Table 3 presents the descriptive statistics for all the variables. We can find that the mean value of MCD quality score was 10.81 out of the maximum 18 (60.06%). The lowest MCD quality score is 7 while the highest is 16. The mean value of Tobin’s Q is 2.044. The lowest Tobin’s Q is 0.211 while the highest is 8.335.

**TABLE 3:** Descriptive statistics.

Variable	Obs	Mean	Std. Dev.	Min	Median	Max
*TobinQ*	1087	2.044	1.581	0.211	1.624	8.335
*Board*	1087	10.64	2.578	6	11	16
*Share*	1087	0.340	0.132	0.100	0.327	0.704
*Size*	1087	9.514	0.453	8.717	9.467	10.68
*SOE*	1087	0.355	0.479	0	0	1
*Lev*	1087	0.418	0.208	0.0467	0.414	0.911
*Growth*	1087	0.117	0.359	−0.401	0.0628	2.601
*CEO-dual*	1087	0.251	0.434	0	0	1
*MCD*	1087	10.81	2.321	7	11	16 (maximum: 18)

The pair-wise correlation between variables was examined using the Pearson Correlation coefficient. The results are presented in Table 4. It can be observed that there are no severe multicollinearity problems between the variables since most coefficients are under 0.5. Furthermore, we can find that the correlation between the independent variable (*MCD*) and dependent variable (*TobinQ*) is statistically significant and positive. Hence, the results support hypothesis 1 that there is a significant and positive association between MCD quality and firm value for Chinese firms.

**TABLE 4:** Results for pair-wise correlation analysis.

	TobinQ	MCD	Board	Share	Size	SOE	Lev	Growth	Duality
*TobinQ*	1								
*MCD*	0.366***	1							
*Board*	−0.204***	−0.118***	1						
*Share*	−0.118***	−0.076**	−0.120***	1					
*Size*	−0.564***	−0.248***	0.281***	0.163***	1				
*SOE*	−0.273***	−0.151***	0.178***	0.126***	0.263***	1			
*Lev*	−0.478***	−0.249***	0.132***	0.063**	0.534***	0.300***	1		
*Growth*	0.017	0.029	0.044	0.046	0.057*	−0.136***	–0.001	1	
*Duality*	0.022	0.017	−0.083***	–0.009	−0.084***	−0.234***	−0.087***	0.032	1

***, **, and *, represent statistical significance at the 1, 5, and 10% level, respectively.

### Multiple Regression Analysis

Results for multiple regression analysis are shown in column 1 in Table 5. It can be found that there is a significant and positive relationship between MCD quality and firm value (*p* < 0.01). The empirical evidence supports hypothesis 1. It suggests that the implementation of the IR approach for corporate reporting would enhance firm valuation significantly. The result of MCD quality is in line with previous studies, such as Lee and Yeo (2016) and Barth et al. (2017).

**TABLE 5:** Results for multiple regression analysis.

	(1)	(2)	(3)	(4)
	*TobinQ*	*TobinQ_new*	*TobinQ*	*TobinQ*
*MCD*	0.225*** (3.58)	0.232*** (3.27)		0.305*** (3.38)
*MCD_new*			0.257*** (3.97)	
*Board*	−0.013 (−0.58)	−0.0182 (−0.74)	−0.012 (−0.56)	−0.027 (−0.77)
*Share*	0.219 (0.88)	0.0642 (0.23)	0.175 (0.70)	0.354 (0.84)
*Size*	−1.805*** (−20.39)	−1.935*** (−19.41)	−1.774*** (−20.03)	−1.816*** (−7.97)
*SOE*	−0.289*** (−3.97)	−0.360*** (−4.40)	−0.268*** (−3.68)	−0.300** (−2.53)
*Lev*	−1.207*** (−6.56)	−1.328*** (−6.40)	−1.124*** (−6.06)	−1.524*** (−3.17)
*Growth*	0.297*** (3.31)	0.371*** (3.67)	0.282*** (3.15)	0.283** (2.00)
*Duality*	−0.194*** (−2.60)	−0.241*** (−2.87)	−0.184** (−2.46)	−0.306** (−2.27)
*Year*	Control	Control	Control	Control
Constant	18.95*** (24.98)	20.45*** (23.93)	18.61*** (24.36)	20.072***(10.09)
Observations	1087	1087	1087	586
Adjusted *R*^2^	0.5749	0.5634	0.5760	0.572

*** and ** represent statistical significance at the 1% and 5% level, respectively. The corresponding t-value for the variable is in the bracket.

### Robustness Tests

In order to gauge the robustness of the main findings, several additional tests were conducted. Firstly, we use substitutional measures for some key variables. The total asset in Tobin’s Q was substituted by the total assets at the end of the year minus the sum of goodwill and intangibles. The substitutional variable is re-labeled “TobinQ_new.” We then run the regression model. As shown in column 2 in Table 5, the empirical results have no substantial changes.

To measure MCD quality from an unbiased perspective, we reassess MCD quality using a new scoring system and rerun the regression model. Specifically, in the new scoring system, we were not constrained by the checklist approach used in the conventional scoring process (counting the presence or the absence of a disclosure item), given the checklist approach brings potential bias (Zhou et al., 2017). In contrast, in order to circumvent such a limitation resulting from the checklist approach, we evaluate MCD quality based on the impression of the coders on whether the disclosed capitals are reported consistently with the guiding principles of IIRF. If a firm discloses MCD in a balanced, materiality-oriented, consistent and comparable, connected, stakeholder-oriented, strategic-focused and future-oriented fashion, the firm obtains the highest MCD score. The results and the main finding are qualitatively similar, continuously reporting a positive association between MCD quality and firm value (see column 3 in Table 5). We also use a propensity score matching (PSM) approach to create treatment (i.e., firms with high MCD quality score) and control (i.e., firms with low MCD quality score) groups, which mitigate the self-selection bias that arises from observed heterogeneities. The results of the PSM analysis (column 4 in Table 5) are qualitatively identical to the non-matched sample. In particular, the coefficient for MCD is 0.226 (*p* < 0.01), consistent with columns 1 of Table 5, Panel A.

### Additional Analysis

In the main analysis, we determine whether IR increases firm value. The following two additional analyses provide opportunities for further understanding the two channels of the market effect through which IR enhances firm value: a better information environment for (1) non-financial stakeholders that allow them to have faith in collaborating with the firm, which can improve profitability and (2) for investors that enable them to estimate stock price more accurately (Barth et al., 2017; Esch et al., 2019; Flores et al., 2019). IR connects a firm’s financial information with previously disconnected non-financial information such as its human and natural capitals. For non-financial stakeholders, they are more likely to “choose to partner with, patronize, or work for” firms with higher transparency in non-financial capitals, and therefore, such firms show an increase in financial performance (Salvi et al., 2021a). For instance, high-quality information on human capital could attract better employees. As a result, these employees can create higher revenues and financial performance for the firms that provide high-quality information on human capital (Salvi et al., 2021b). IR can also allow shareholders better understand how the non-financial performances and operations of a firm impact its financial performance, thus benefiting “shareholders because they reduce the information asymmetry concerning the impact of non-financial information on financial performance” (Akisik and Gal, 2019, p. 5). Thus, the adoption of an IR approach improves the usefulness of financial information for investors.

### Multiple Capitals Disclosure Quality and Decision-Making of Non-financial Stakeholders

Using the IR approach would improve the relationship between the firm and non-financial stakeholders, and consequently, the firm can obtain support from stakeholders. The support of stakeholders may include the provision of various capitals, such as human capital and social capital (Heugens and Lander, 2009; De Villiers et al., 2014b). If companies manage these capitals efficiently and effectively, it will certainly improve the financial benefits of firms. The income of a firm that uses IR approach will increase as stakeholders are more willing to collaborate with such a firm and therefore, they buy more products or services of the firm. According to Barth et al. (2017, p. 48), “disclosure about the six capitals can be informative to stakeholders, such as customers and employees, who associate with more socially responsible firms. This can result in increased financial performance.” Thus, we examine the association between MCD quality and profitability (also using a lead-lag model). Table 6 demonstrates the regressions of profitability on MCD quality. The results show that the association between MCD quality and profitability is significantly positive at the 1% level. Thus, this additional analysis provides preliminary evidence suggesting IR can enhance a firm’s profitability.

**TABLE 6:** MCD disclosure and profitability.

	ROA
*MCD*	0.009*** (3.49)
*Board*	0.001 (0.54)
*Share*	−0.006 (−0.64)
*Size*	0.024*** (6.63)
*SOE*	−0.001*** (−3.40)
*Lev*	−0.146*** (−19.63)
*Growth*	0.031*** (8.51)
*Duality*	−0.005 (−1.63)
*Year*	Control
Constant	−0.128*** (−4.18)
Observations	1087
Adjusted R^2^	0.3457

*** represents statistical significance at the 1% level, respectively.

### Multiple Capitals Disclosure Quality and Decision-Making of Investors

We are also interested to see whether a firm with a higher MCD quality has a greater value relevance of financial information relative to firms with a lower MCD quality. Drawing on prior research (e.g., Baboukardos and Rimmel, 2016; Li, 2017; Cortesi and Vena, 2019), we adopted the price-model of Ohlson (1995) to examine if MCD quality by Chinese companies could have a positive impact on the value relevance of financial reporting. The price model often refers to the value of the adjusted R-squared to assess the explanatory power of financial information on the share price (namely, the value relevance of financial information). We categorized the sample companies into two groups in that the one included the companies whose MCD quality score was in the top 50% of the sample, and the other one was otherwise. We then conducted a regression analysis using the price model of Ohlson (1995). We can find that the adjusted R-squared for group 1 (the top 50%) is greater than the other group (see Panel A of Table 7^4^), which indicates that there is a positive association between MCD quality by Chinese firms and the value relevance of financial information. To assess the robustness of the results, we further divided the sample companies into four groups based upon the MCD quality score, in terms of 25% percentile. Then we run the price model of Ohlson (1995). The results, as shown in Table 7, reveal that the adjusted R-squared for the top 25% of sample companies is the greatest, whereas that of the last 25% is the poorest. Therefore, the findings are consistent in general (see Panel B of Table 7) and are in line with the findings of Li (2017) and Cortesi and Vena (2019).

**TABLE 7:** MCD disclosure and value relevance of financial information.

Panel A	
**Group**	**Adj. *R*^2^**

Top 50%	0.3343
The other 50%	0.3012

**Panel B**	
**Total score**	**Adj. *R*^2^**

Top 25%	0.3498
25–50%	0.2871
50–75%	0.2904
The rest 25%	0.2630

## Discussion and Conclusion

Our paper examines the association between IR quality and firm value, using a sample of Chinese listed A-share companies. The results reveal that there is a significant and positive relationship between IR quality by Chinese firms and firm value, indicating the IR approach affects the pricing-decision making of investors. Also, we find that IR quality has an impact on the decision-making of non-financial stakeholders and helps investors to make better decision-making. Based upon the findings, we would argue that companies would be greatly benefited if they adopt an IR approach for corporate reporting because it can shape stakeholders’ perceptions of the organization. This is consistent with the expectation of IIRC to develop and promote IR worldwide (IIRC, 2013).

IR approach can bring an increase in accountability and transparency in corporate reporting (Owen, 2013). In response to the information needs of a broad range of stakeholders, IR provides non-financial information that is of interest to non-financial stakeholders to enhance accountability toward them (Steenkamp, 2018). The emergence of IR also responds to society’s demands for greater transparency in terms of organizational performance. According to Beyne et al. (2021), IR approach promotes the achievement of 17 Sustainable Development Goals of the 2030 Agenda for Sustainable Development. Furthermore, adopting IR approach is believed to be associated with higher financial reporting quality (Owen, 2013). On the one hand, as indicated by this study’s findings, IR approach enhances value relevance of financial information. On the other hand, as found by Lemma et al. (2019), IR approach ensure the reliability of financial information. Prepares have less opportunity to manipulate financial information and the management has lower pressures to make earnings management for pursuing short-term performance targets. Additionally, IR also contributes to the efficiency of non-financial reporting. IR is recognized as being different from sustainability reporting in treating non-financial information. Sustainability reporting purely puts emphasis on environmental and social impacts (Minutiello and Tettamanzi, 2021) whereas IR explicitly treats these non-financial aspects of a firm as the capital. IR’s prominent display of non-financial performance to the creation of value is commendable as it is important to investors and other stakeholders to accurately assess a firm’s value and performance.

This study has some implications for researchers, corporate management, investors, regulators and relevant policymakers. First, as an initial study with regard to IR quality by Chinese firms, we expect that it would induce more researchers in the area to conduct empirical research in different environments, so as to advance the development of IR related theories and practices. Second, the perspective of behavioral decision theory in the context of IR focuses on the decision usefulness of IR and the decision to implement IR (Velte and Stawinoga, 2016). The empirical evidence of this study suggests that IR would bring benefits to the decision-making of stakeholders, leading to benefits for the firms as a result. Thus, the management of a firm could take advantage of the empirical evidence of this study to make decisions regarding the adoption and implementation of IR in the firm. As a consequence, it would facilitate the adoption and implementation of IR widely globally. Third, investors and non-financial stakeholders such as government officials, policymakers could obtain some insights from the findings of this study, which could help them make better recommendations and decisions. Fourth, IR also brings the transformation of KPIs system of companies from a traditional one that overly focuses on financial performance indicators provided by the financial reporting to a new framed one that concerns the indicators in terms of six capitals (Albertini, 2019; Almăşan et al., 2019). Non-financial KPIs are seldomly based on generally accepted accounting principles (Maniora, 2015). For instance, generally accepted accounting principles do not require the recognition of intangible assets in financial reporting (Salvi et al., 2021b). Critics of the traditional KPIs system claim that financial performance indicators could not adequately represent what a firm is all about (Argento et al., 2019). Thus, managers are encouraged to restructure their KPIs system in accordance with IR approach. According to Dilling and Caykoylu (2019, p. 2). Such a restructured KPIs system “can range from customer satisfaction metrics to stakeholder engagement key performance indicators (KPIs), from energy savings and carbon footprint numbers to dollar figures for investment in innovations, and from return to shareholders to social value input or investment in property, plant, and equipment (PPE).”

This paper is subject to some limitations that may offer avenues for further research. First, it focuses on the environmentally sensitive sectors only because this paper is subject to the difficulty of manual content analysis toward substantial numbers of corporate reports provided by a large sample of companies (Marrone and Oliva, 2019; Manes-Rossi et al., 2021). Future research could expand the sample size by using machine-learning approaches. Second, this study mainly examines the effect of IR quality on firm value and two channels of the market effect through which IR enhances firm value. Future research could be extended to investigate other channels through which IR could affect firm value, such as the mitigation of agency problems or the improvement of internal decision-making. Third, we are calling for more research on market perceptions of IR to contribute to the application of behavioural decision theory on group decision making.

## Data Availability Statement

The original contributions presented in the study are included in the article/supplementary material, further inquiries can be directed to the corresponding author.

## Author Contributions

YS and YA conceived and designed the study and drafted the manuscript. XQ contributed to the acquisition, analysis, and interpretation of data for the work. QF guided the research and revised it critically. NW provided thoughtful and constructive suggestions and made the preliminary proofreading.

## Conflict of Interest

The authors declare that the research was conducted in the absence of any commercial or financial relationships that could be construed as a potential conflict of interest.

## Publisher’s Note

All claims expressed in this article are solely those of the authors and do not necessarily represent those of their affiliated organizations, or those of the publisher, the editors and the reviewers. Any product that may be evaluated in this article, or claim that may be made by its manufacturer, is not guaranteed or endorsed by the publisher.

## Footnotes

1 In this paper, multiple capitals disclosure quality refers to the level of consistency between corporate reporting by Chinese listed firms and the self-constructed framework.

2 All companies listed in China must apply Chinese Accounting Standards for the preparation of their financial reporting. Chinese listed firms disclose non-financial information, such as information with regard to two social-oriented types of capital (human, social and relationship capitals), intellectual capital, and one environmental-oriented type of capital (natural capital), in stand-alone CSR/sustainability/ESG/Environment reporting and/or in the management discussion and analysis (MD&A) section of firms’ annual reports. However, the initiatives for mandatory and voluntary disclosure of non-financial reporting have coexisted in China (Shen et al., 2020; Shaheen et al., 2021; Wu and Hąbek, 2021). There are five main instruments on mandatory disclosure in mainland China, including the Shanghai Stock Exchange (SHSE): *Notice of Doing a Better Job for Disclosing 2008 Annual Reports*; the Shanghai Stock Exchange: *Guidelines for Environmental Information Disclosure of Listed Companies*; the China Securities Regulatory Commission: *Regulations on Information Disclosure of Listed Companies*; the Shenzhen Stock Exchange: *Notice of Doing a Better Job for Disclosing 2008 Annual Reports*; and *the Notice of the China Securities Regulatory Commission on Promulgating Standards Concerning the Contents and Formats of Information Disclosure by Companies Offering Securities to the Public No.2—Contents and Formats of Annual Reports (2017 Revision)*. For instance, the SHSE mandated CSR reporting for companies included in the SHSE Corporate Governance Index, companies that issue overseas-listed foreign shares, and companies in the financial industry. Currently, the guidelines followed voluntarily by some Chinese companies in compiling their non-financial reporting are mainly the GRI G4 guidelines, GRI standards and the Corporate Social Responsibility Report Compilation Guide released by the Corporate Social Responsibility Research Centre of the Chinese Academy of Social Sciences.

3 Special treatment (ST) was introduced by the Shanghai and Shenzhen Stock Exchanges in April 1998. If a listed company has two consecutive years’ financial losses or is technically insolvent, it would be labeled as an ST company. Eliminating ST companies from a sample is common in prior research related to the Chinese stock markets since their financial indicators are usually abnormal.

4 According to the price model, the value of adjusted R-squared can be used to evaluate the explanatory power of financial information on the share price (namely, the value relevance of financial information). The purpose of the price model is to examine the value relevance of financial information, and for this purpose, only the results with respect to the Adj.*R*^2^ for the two groups are relevant, whereas the detailed regression results of the model are not much relevant to the purpose of the study. However, detailed regression results can be obtained if required.
